# Effect of Organic Food Intake on Nitrogen Stable Isotopes

**DOI:** 10.3390/nu12102965

**Published:** 2020-09-28

**Authors:** Olivier L. Mantha, Maya Laxmi Patel, Régis Hankard, Arnaud De Luca

**Affiliations:** Faculté de Médecine, Université de Tours, INSERM, N2C UMR 1069, 37032 Tours, France; maya.patel@inserm.fr (M.L.P.); regis.hankard@inserm.fr (R.H.); arnaud.deluca@univ-tours.fr (A.D.L.)

**Keywords:** nitrogen-15 isotopic abundance, organic food intake, animal and plant protein intake

## Abstract

Food choices affect the isotopic composition of the body with each food item leaving its distinct isotopic imprint. The common view is that the natural abundance of the stable isotopes of nitrogen (expressed as δ^15^N) is higher in animals than in plants that constitute our contemporary diets. Higher δ^15^N is thus increasingly viewed as a biomarker for meat and fish intake. Here we show that organic compared to conventional farming increases plant δ^15^N to an extent that can appreciably impact the performance of δ^15^N as a biomarker. The error that can arise when organic plants are consumed was modelled for the entire range of proportions of plant versus animal protein intake, and accounting for various intakes of organic and conventionally grown crops. This mass balance model allows the interpretation of differences in δ^15^N in light of organic food consumption. Our approach shows that the relationship between δ^15^N and meat and fish intake is highly contextual and susceptible to variation at the population, community or group level. We recommend that fertilization practices and organic plant consumption must not be overlooked when using δ^15^N as a biomarker for meat and fish intake or to assess compliance to nutritional interventions.

## 1. Introduction

Biomarkers for the intake of plant and animal proteins can help us to understand relationships between diet and health, and to assess adherence to nutritional interventions aiming at modulating reliance on animal and plant proteins. The natural abundance of the stable isotopes of nitrogen (δ^15^N) is increasingly viewed as a biomarker for meat and fish intake [1,2,3,4,5]. Recent controlled feeding studies found associations between tissue δ^15^N and meat and fish intake [1,2], reinforcing the potential of δ^15^N as a dietary intake biomarker.

The rationale behind this approach lies on the fact that organisms are enriched in ^15^N relative to their diet [6] likely due to isotopic fractionation during certain metabolic reactions [7]. Animals are, thus, enriched in ^15^N compared to the plants and other animals that they consume, an isotopic offset that varies with changes in protein and amino acid metabolism [8,9]. Due to the greater length of aquatic food chains, marine products and fish, particularly when not farm-raised [10], have the highest ^15^N content [11]. δ^15^N is, therefore, a biomarker of meat and fish intake under the premise that the animal proteins consumed have a higher ^15^N content than the plant proteins consumed. The ^15^N content depends on the nitrogen cycle from which a product originates with nitrogen conversion processes fractionating isotopes to different degrees depending on the context [12,13,14]. Human activities and agricultural practices affect the nitrogen cycle.

This higher ^15^N isotopic abundance in animals than in plants is, indeed, expected in nature. Human mastering of agriculture might have strengthened this difference with the use of synthetic fertilizers. Synthetic fertilizers, an important nitrogen source in modern agriculture, have a low ^15^N content (δ^15^N close to 0 ‰) because their nitrogen is derived from atmospheric N_2_ and fractionation tends to be minimal during their production [12,15]. However, in recent years, the market for organic food has been increasing and land use for organic agriculture expanding [16]. Synthetic fertilizers are prohibited in organic farming, where fertilization is achieved with the application of natural products such as animal manures. According to Bateman and Kelly [13], fertilizers that may be permitted in organic agriculture have a higher ^15^N content (mean δ^15^N of 8.5 ‰). Many studies have consequently shown a higher δ^15^N in organically compared to conventionally grown crops [14] suggesting that the δ^15^N of the animal proteins compared to the plant proteins consumed, may not be as high when organic plants are the preferred choice.

This study assesses the impact that organic food and fertilization practices can have on the performance of δ^15^N as a biomarker. A nitrogen mass balance model was developed to test the effect of organic plant intake on body proteins δ^15^N at various relative intakes of plant and animal proteins. Various scenarios were identified where the performance of δ^15^N as a biomarker for fish and meat intake is impacted differently.

## 2. Materials and Methods

### 2.1. ^15^N Natural Isotopic Abundance of Organic Plants

δ^15^N values of edible plants detailed in Supplementary Materials Table S1 were obtained from published literature. As there are more published δ^15^N data for conventionally grown plants, only studies containing data for both crops considered as organic and conventionally grown were included in our analysis to avoid having imbalanced data in terms not only of plants categories per farming method, but also of sample size. This resulted in the inclusion of 21 studies in our analysis (with 80 and 90 δ^15^N values for conventionally and organically grown crops, respectively). The isotopic offset between organic and conventionally grown plants (Δ^15^N_org-conv_) was determined from the median δ^15^N values of the two farming methods:

Δ^15^N_org-conv_ = δ^15^N_organic plants_ − δ^15^N_conventional plants_ (1)

### 2.2. Effect of Organic Plant Protein Intake on Body ^15^N Natural Isotopic Abundance

A two-sources isotope mixing model can be use to relate the proportions of plant to animal proteins consumed to tissue δ^15^N if the isotopic offset between tissue and dietary proteins owing to metabolism is considered. Human tissues δ^15^N equals the sum of the δ^15^N of the different proteins consumed, weighted by their proportion to dietary nitrogen, plus an isotopic discrimination factor that is linked to amino acid and protein metabolism [17,18]. As detailed in Supplementary Materials Text S1, separating the nitrogen consumed into nitrogen from either animal or plant sources and further separating the plant sources into conventionally and organically grown plants, the impact of organic food intake on tissue protein δ^15^N can be assessed using a simple isotopic mass balance calculation:

δ^15^N_tissue proteins_ = δ^15^N_animal_ × P_animal_ + (δ^15^N_conv plants_ + Δ^15^N_org-conv_ × P_org_) × (1-P_animal_) + Δ^15^N_tissue-diet_ (2)

where δ^15^N_tissue proteins_ is the isotopic composition of the body proteins, δ^15^N_conv plants_ and δ^15^N_animal_ are the δ^15^N values of dietary plant and animal proteins, respectively. P_animal_ is the proportion of dietary proteins occupied by animal proteins and 1-P_animal_ is the proportion occupied by plant proteins (where protein refers to protein nitrogen). P_org_ is the proportion of dietary plant proteins occupied by proteins from organically grown plants. Δ^15^N_tissue-diet_ is the isotopic change between tissue and dietary proteins owing to metabolism, set here at 3.5‰ (an approximation based on the liver proteins analyzed after the consumption of two diets with different effects on amino acid metabolism [17]).

The error resulting from organic plant intake on δ^15^N_tissu__e proteins_ was then calculated as follows:

δ^15^N absolute error = Δ^15^N _org-conv_ × P_org_ × (1-P_animal_) (3)

δ^15^N relative error = 100 × δ^15^N absolute error/[δ^15^N_animal_ × P_animal_ + δ^15^N_conv plants_ × (1-P_animal_) + Δ^15^N_tissue-diet_] (4)

δ^15^N_tissue proteins_ and its absolute and relative error were computed over the whole range of P_animal_ (0 to 1) and for each 0.1 (10%) increment in P_org_. Calculations were performed over the whole range of δ^15^N values observed in animal proteins according to Huelsemann et al. [11], with the lowest end of δ^15^N_animal_ at 2,83‰, the average δ^15^N of poultry, and the highest end of δ^15^N_animal_ at 12.39‰, the average δ^15^N of fish. As in Equation (1), δ^15^N_conv plants_ was set as the median of the literature values for conventionally grown plants.

## 3. Results

### 3.1. ^15^N Natural Isotopic Abundance of Organic Plants

**Figure 1:**
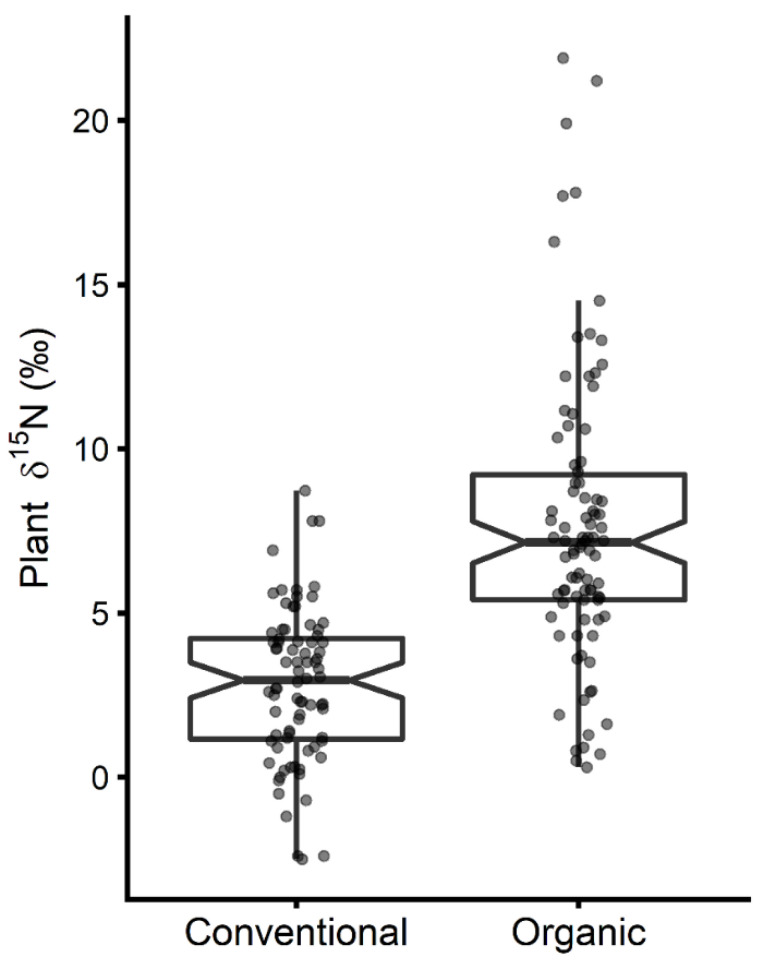
Reported plant δ^15^N values for conventional and organic farming (Table S1). The box represents the interquartile range (IQR), the dark line represents the median, whiskers extend to 1.5 times the IQR, and notches give an estimate of the 95% confidence interval around the median.

Literature δ^15^N data for plants grown conventionally or using organic fertilization are displayed in Figure 1. Crops fertilized organically tended to have a higher δ^15^N. The mean δ^15^N of organically grown plants was 7.7 ± 4.4‰ (median = 7.2‰) while it was 2.8 ±2.3‰ (median = 3.0‰) for plants fertilized inorganically resulting in a Δ^15^N_org-conv_ of 4.2‰. Figure 1 shows an overlap between the δ^15^N values of organically and conventionally grown plants. The lowest δ^15^N value for conventional farming was observed in a batch of tomatoes and was of −2.5‰ [15] while the highest value, 8.72‰, was observed in peppers [19] (Table S1). For organic agriculture, the lowest δ^15^N value, 0.3‰, was measured in peas obtained from an organic grocery store [20] and the highest δ^15^N values, 21.89‰, in lettuce fertilized with bat guano [21] (Table S1).

### 3.2. Effect of Organic Plant Protein Intake on Body ^15^N Isotopic Abundance

Model output (δ^15^N_tissue proteins_, Equation (2)) is shown in Supplementary Materials Figure S1. At the low end of the δ^15^N_animal_ spectrum, δ^15^N_tissue proteins_ increases not only with organic plant intake (Figure S1A), but also with plant protein intake. The opposite relationship between δ^15^N_tissue proteins_ and plant protein intake is seen at the high end of the δ^15^N_animal_ spectrum (Figure S1B).

**Figure 2:**
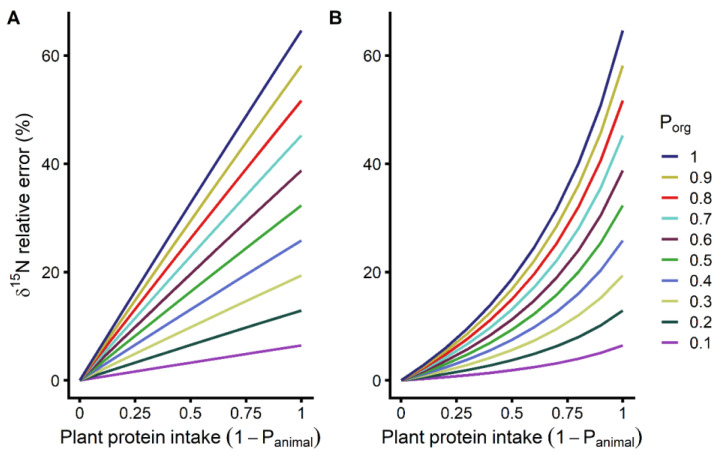
δ^15^N relative error resulting from organic plant intake, as a function of proportional plant protein intake (1-P_animal_) and organic plant intake (P_org_). Data are presented for both the low ((**A**), 2.83‰) and high ((**B**), 12.39‰) ends of the animal proteins δ^15^N range.

δ^15^N relative error introduced by organic plant consumption is plotted in Figure 2 as a function of the contribution of plant proteins to total protein intake (1-P_animal_). Figure S2 shows the absolute error. As seen from Equation (3), the absolute error is independent of δ^15^N_animal_. At the usual level of plant protein intake observed in the French and American populations (~30% of protein from plant sources, 1-P_animal_ = 0.3; [22,23,24]), the error ranged from 0.1‰ to 1.3‰ for low to high P_org_ (0.1 to 1).

Figure 2 shows the relative error for both the low (Figure 2A) and high (Figure 2B) ends of the δ^15^N_animal_ spectrum. When δ^15^N_animal_ of the dietary intake is low, a situation where the major source of animal protein is poultry, δ^15^N relative error seems to vary linearly with the relative importance of plant proteins to total protein intake (Figure 2A). In this situation, at the usual level of plant protein intake in the French and American populations (~30% of protein from plant sources, 1-P_animal_ = 0.3; [22,23,24]), δ^15^N relative error ranged from 2.0% to 19.8% for low to high P_org_. In contrast, Figure 2B shows that when δ^15^N_animal_ of the dietary intake is high, a situation where the main source of animal protein is fish or marine products, δ^15^N relative error vary non-linearly with the relative importance of plant proteins to total protein intake. In this case, at the usual level of plant protein intake, δ^15^N relative error ranged from 1.0% to 9.6% for low to high P_org_. Maximal error occurs when all of the plant protein intake is from organic sources (P_org_ = 1), and the proportional plant protein intake is high (1-P_animal_ close to 1). In that context, for both the low (Figure 2A) and high (Figure 2B) ends the δ^15^N_animal_ spectrum, the maximal δ^15^N absolute error is 4.2‰ and the relative error 64.6%. As shown in Equation (4), relative error was not only a function of δ^15^N_animal_, but also dependent on Δ^15^N_tissue-diet_.

## 4. Discussion

Whereas natural ^15^N abundance is a candidate biomarker for meat and fish intake [1,2] due to a higher ^15^N content in meat and fish than in plants, it has been shown that organic compared to conventional farming increases plant δ^15^N [14]. The present study demonstrates that this isotopic particularity of organic plants impacts the performance of δ^15^N as a biomarker in humans, for meat and fish intake. Proportional changes in organic compared to non-organic plant consumption impact the body δ^15^N differently depending on plant and animal protein intakes, and on the δ^15^N of the animal proteins consumed.

### 4.1. Organic Farming Impacts Plant Nitrogen Isotopic Composition

Plant δ^15^N was lower on average for conventionally grown plants than for organically grown plants (Δ^15^N_org-conv_ = 4.2‰, Figure 1) due to the use of fertilizers with a high ^15^N content such as animal manures in organic agriculture. Huelsemann et al. [11] reported a similar yet slightly lower average difference of 3.7‰ between vegetables grown conventionally versus organically with a δ^15^N of 6.8‰ in organic plants. In the present study, organic plants had an average δ^15^N of 7.7‰, a value higher than the average δ^15^N of many animal products. It must be emphasized that these average isotopic differences between conventionally and organically grown plants are highly contextual and dependent on the nitrogen cycle from which the products originate. It has been shown, for instance, that organic fertilization with green manure alone instead of animal manure can result in lower plant δ^15^N [25,26]. Local practices and potentially rapidly evolving national regulations on organic fertilization must be taken into account when assessing the impact of organic farming on plant δ^15^N, and thus on body δ^15^N as a biomarker for fish and meat intake in a population, or a community, and as a marker of compliance in study participants.

### 4.2. Organic Plant Intake Affects the Performance of δ^15^N as a Biomarker

We demonstrate here that organic plant intake can affect the performance of ^15^N natural isotopic abundance as a biomarker for meat and fish intake. This idea was illustrated by computing δ^15^N changes introduced by organic plant consumption using a mass balance model accounting for proportional intakes of plant and animal proteins (Figure 2). Various proportional intakes of organic relative to non-organic plants were also considered to simulate different frequencies of purchasing organic foods. The goal of this approach was, rather than to reflect the average behaviour of a population, to explore all possibilities that could be seen among individuals and between different populations and communities.

More precisely, potential behavioural differences between individuals affecting their tissue δ^15^N that were studied were: (1), the intake of plant relative to animal proteins (1-P_animal_), (2) the intake of organic relative to non-organic plant proteins (P_org_), and (3) the δ^15^N of animal protein intake (Figure 2A vs. Figure 2B), which depends on the types of animals consumed.

Figure 2 shows that the impact of organic plant consumption on tissue δ^15^N depends on the intake of plant relative to animal proteins. Studies of the French and American populations showed that plant proteins represent, on average, around 30% of the proteins consumed (1-P_animal_ = 0.3) [22,23,24]. The proportion of plant protein intake is at 100% on a vegan diet, and when organic plants are consumed, this has the greatest impact on tissue δ^15^N.

Although the effect of organic farming on the δ^15^N of vegetables was undeniable in a previous study, it was argued that organic plant intake has a minor effect on the body δ^15^N of contemporary Germans, who obtains few of their proteins from vegetables and around 30% from cereals, as no significant difference between conventionally and organically grown cereals were observed [11]. There is, however, data clearly showing that organic fertilization can impact cereals δ^15^N [25,26,27], indicating that such a generalization may not be possible.

We acknowledge some limitations of the current approach. The first one lies in the estimation of Δ^15^N_org-conv_ since very little data are available for some plant categories like legumes that tend to have a low δ^15^N due to atmospheric N_2_ fixation. A smaller or minor effect of organic fertilization could thus be expected in these plants. Nonetheless, legumes account for a small proportion of total plant protein intake in some populations [24]. This shows that Δ^15^N_org-conv_ and, therefore, the effect of organic plant intake on δ^15^N, is highly circumstantial and dependent on the nitrogen cycle from where consumed plants are grown. Furthermore, the lower digestibility of some vegetable proteins was not explicitly accounted for in Equation (2), which means that proportional plant protein intake (1-P_animal_) as used here is the contribution of effectively digested plant proteins to total effectively digested proteins. 

### 4.3. Animal Protein Sources Affect the Performance of δ^15^N as a Biomarker

The source of animal protein is another modulator of the performance of δ^15^N as a biomarker that was studied. There is a well-documented δ^15^N spectrum between different animal categories with poultry at the lower end and fish at the higher end of the δ^15^N values and with considerable δ^15^N variability within the animal categories [10,11]. While consumers that choose organic plants might also prefer organic meat, there appear to be no clear effects of organic animal production on their δ^15^N, which must also be considered as highly context dependent [28]. At the two ends of this δ^15^N spectrum of animal products, δ^15^N behave in opposing ways with changes in organic plant intake. When organic plants are consumed and poultry is the main source of animal proteins, increasing proportional intake of plant proteins makes the body δ^15^N closer to that of meat and fish, while it is the opposite when animal proteins are predominantly from an aquatic origin (Figure S1). This reemphasizes that the capacity of δ^15^N as biomarker for meat and fish intake [1,2] is very contextual. Furthermore, these opposite δ^15^N behaviors between the two modeled dietary scenarios (Figure S1) show that the directionality of the error that ranges between 0‰ and Δ^15^N_org-conv_ (Figure S2, Equation (3)) depends on the difference in δ^15^N between dietary plants and animals. If the δ^15^N is higher in dietary plants than in animal proteins, the error is positive; when δ^15^N of animal proteins is higher the error is negative. Opposite error directionalities show that the common view that meat intake increases tissue δ^15^N is not always valid. Although, as can be seen from Equation (3), the absolute isotopic impact in ‰ of organic plant intake (Figure S2) is independent of the source of animal proteins, δ^15^N relative error propagates differently along the plant protein intake axis depending on the δ^15^N of the animal proteins consumed (Figure 2). Having poultry as the main source of animal proteins results in a higher relative impact at low proportional intake of plant proteins (Figure 2A). In contrast, if the main source of animal proteins is from an aquatic origin, the relative impact on δ^15^N of organic plant intake will increase more sharply when the proportional intake of plant proteins is high (non-linear increase in Figure 2B). This latter scenario is of relevance to pescatarians on a predominantly plant-based diet supplemented with a small amount of fish.

Different relative error propagations due to varying sources of animal proteins raises the question of the meaning of the relative error for an isotopic biomarker. According to our model predictions, this might depend on the δ^15^N of the studied population and on its homogeneity in terms of δ^15^N. Relative error could reflect the effect size of the relationship between animal protein intake and δ^15^N, which can differ across populations due to different δ^15^N values of the animals and plants consumed. This should be explored more deeply in future work and pertains to another caveat of our approach which is that the isotopic offset between tissue and dietary proteins (Δ^15^N_tissue-diet_ in Equation (2)) is likely to increase with plant protein intake [17] due to changes in amino acid metabolism. In proportion, metabolically induced δ^15^N variations have different impacts depending on the δ^15^N of consumed proteins.

## 5. Conclusions

Natural differences in nitrogen isotopic abundance between individuals, is increasingly viewed as a biomarker for meat and fish intake. With an isotopic mass balance equation, considering proportional animal and plant protein intakes, we show that this is not strictly true when organic plants are consumed. There can even be opposite relationships between tissue ^15^N content and animal protein intake depending on the context. There is thus no systematic relationship between meat and fish intake and human tissue isotopic composition. Both fertilization practices and their potential evolution, and organic plant consumption must be considered when using the natural abundance of the stable isotopes of nitrogen as a dietary intake biomarker or a compliance marker. A case-by-case approach accounting for the nitrogen cycle should be used and adapted to the population, community or individuals concerned. We hope that future implications include a cautious context-dependent use of the natural abundance of nitrogen isotopes as a marker of food choices and adherence to nutritional advice.

## Supplementary Materials

The following are available online at https://www.mdpi.com/2072-6643/12/10/2965/s1: Figure S1: Model output (Equation (2)) showing δ^15^N_tissue proteins_ at the low (A) and high (B) ends of the δ^15^N_animal_ spectrum, Figure S2: δ^15^N absolute error resulting from organic plant intake (Equation (3)), Table S1: Edible plant δ^15^N values, Text S1: Calculations of the effect of organic food intake on tissue proteins δ^15^N.
