# Supplementary material for: Effect of Organic Food Intake on Nitrogen Stable Isotopes
Source: Nutrients. 2020 Sep 28;12(10):2965. doi: 10.3390/nu12102965 (PMC7601395; doi:10.3390/nu12102965)
Supplement: Supplementary file 1 [file nutrients-12-02965-s001.pdf]

nutrients

MDPI

Supplementary Materials

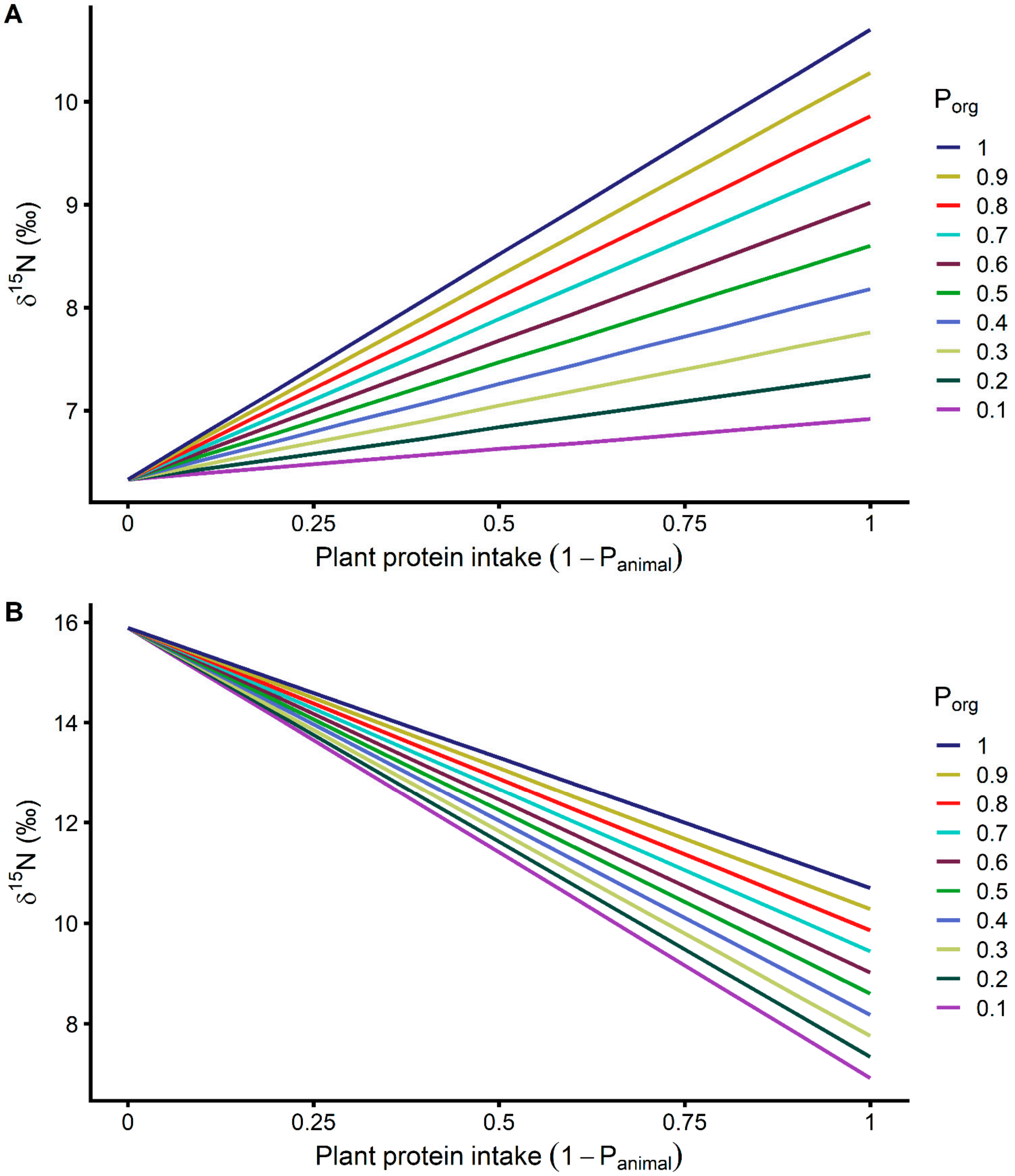

Figure S1. Model output (equation 2) showing $\delta^{15}N_{tissue\ proteins}$ at the low (A) and high (B) ends of the $\delta^{15}N_{animal}$ spectrum. Proportional plant protein intake ($1-P_{animal}$) varies from 0 to 1 with 0.1 increments in proportional organic plant intake (P_{org}) plotted.

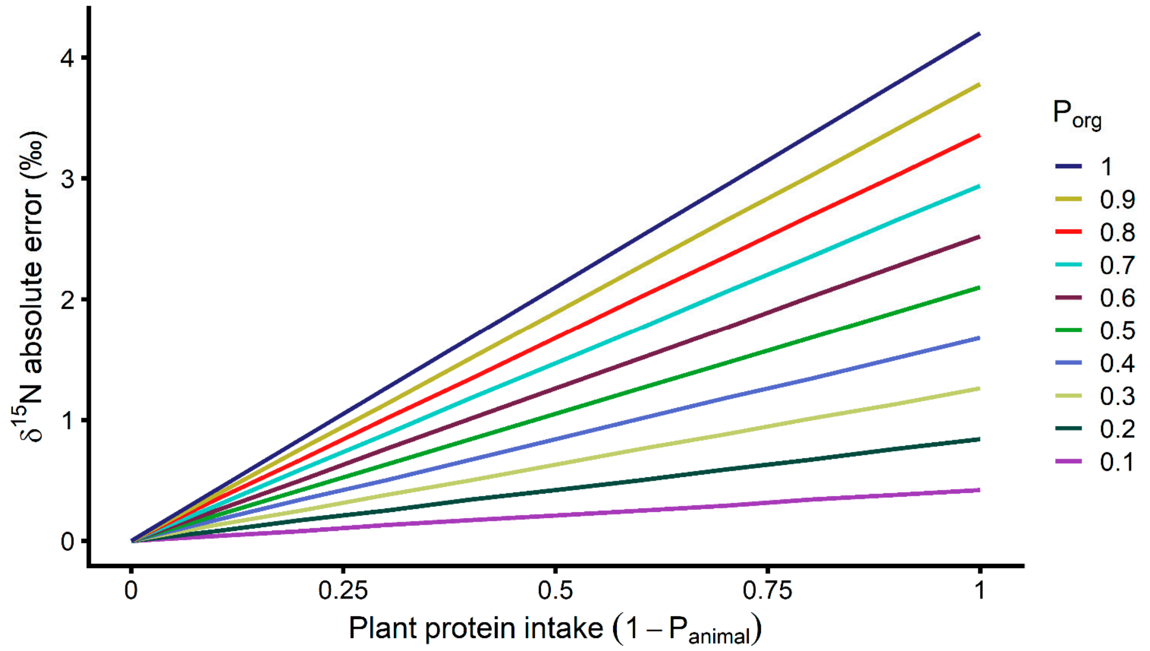

Figure S2. δ^{15}N absolute difference resulting from organic plant intake (equation 3). This absolute error is independent of $\delta^{15}N_{animal}$. Proportional plant protein intake ($1-P_{animal}$) varies from 0 to 1 with 0.1 increments in proportional organic plant intake (P_{org}) plotted.

Table S1. Edible plant δ15N values. The δ15N values were used for estimating $\Delta_{org\text{-}conv}$ in equation 1 and are plotted in Figure 1.

Edible plants	Fertilization type	$\delta^{15}N$	SD	*n*	References
Brassica oleracea L	conventional	3.6	2.9	3	[1]
Brassica oleracea L	organic	16.3	0.4	2	[1]
Broccoli	conventional	4.3		3	[2]
Broccoli	conventional	3.92	2.25	12	[3]
Broccoli	organic	12.2		3	[2]
Broccoli	organic	6.08	1.76	5	[3]
Broccoli	organic	8.96	1.32	9	[3]
Broccoli	organic	12.57	2.81	15	[3]
Cabbage	conventional	5.7			[4] data from [5]
Cabbage	organic	8.7			[4] data from [5]
Carrot	conventional	4.1	2.6	17	[6]
Carrot	conventional	3.9	2.3	5	[7]
Carrot	conventional	3.5	0.4		[8]data from [5]
Carrot	organic	5.7	3.5	13	[6]
Carrot	organic	3.7	1.4	5	[7]
Carrot	organic	6.7	1.0		[8] data from [5]
Cauliflower	conventional	5.5	2.7	3	[7]
Cauliflower	organic	10.7	4.3	3	[7]
Chicory Palla rosa	organic	7.7	1.4	4	[7]
Chicory Palla rosa	conventional	2.0	0.8	3	[7]
Chicory Pan di zucherro	organic	5.3	1.7	3	[7]
Chicory Pan di zucherro	conventional	2.3	1.4	3	[7]
Clementine	conventional	6.9		21	[9] data from [10]
Clementine	organic	7.2		31	[9] data from [10]
Corn	conventional	0.8		3	[2]
Corn	organic	4.8		3	[2]
Cucumber	conventional	2.7		3	[2]
Cucumber	conventional	3.3	1.4	4	[1]
Cucumber	organic	12.3		3	[2]
Cucumber	organic	13.3	2.6	4	[1]

Eggplant	organic	8.5		3	[2]
Eggplant	conventional	4.5		3	[2]
Eggplant	conventional	2.7	3.0	2	[1]
Eggplant	organic	13.4		1	[1]
Endive	conventional	0.6	2.7	3	[7]
Endive	organic	6.9	1.1	5	[7]
Garlic	conventional	2.6	2.6	3	[7]
Garlic	organic	4.3	1.9	3	[7]
Kohlrabi	conventional	7.8	1.3	3	[7]
Kohlrabi	organic	6.2	4.3	3	[7]
Leek	conventional	1.9	1.0	4	[7]
Leek	organic	7.3	3.0	4	[7]
Lettuce	conventional	5.2		3	[11]
Lettuce	organic	9.6		3	[11]
Lettuce	organic	11.9	2.73	30	[3]
Lettuce	conventional	2.9	4.3	55	[6]
Lettuce	conventional	5.3		3	[11]
Lettuce	conventional	1.77	1.71	10	[3]
Lettuce	conventional	2.2			[8] data from [5]
Lettuce	conventional	4.1	0.6	2	[1]
Lettuce	organic	7.6	4.1	49	[6]
Lettuce	organic	8.0		3	[11]
Lettuce	organic	21.89	2.08	9	[3]
Lettuce	organic	5.68	1.17	23	[3]
Lettuce	organic	5.5			[8] data from [5]
Lettuce	organic	13.5	1.0	4	[1]
Maize	organic	8.1	1.6	6	[12]
Maize	conventional	5.8	0.2	6	[12]
Maize	conventional	4.2	1.1	2	[1]
Maize	organic	21.2	0.2	6	[12]
Maize	organic	17.7	4.3	2	[1]
Onion	conventional	5.2	1.8	3	[7]

Onion	organic	6.8	1.6	3	[7]
Orange	conventional	5.5		56	[9] data from [10]
Orange	conventional	4.64	0.35		[13]
Orange	organic	7.6		42	[9] data from [10]
Orange	organic	6.74	0.7		[13]
Orange	organic	8.95	0.38		[13]
Orange	organic	8.45	0.61		[13]
Parsley	conventional	4.5	4.5	3	[7]
Parsley	organic	5.4	1.1	6	[7]
Peach	conventional	0.1		24	[9] data from [10]
Peach	organic	1.9		95	[9] data from [10]
Peas	organic	0.3		3	[2]
Peas	conventional	0.2		3	[2]
Pepper	conventional	-2.4			[14]
Pepper	organic	4.3	0.2	3	[7]
Pepper	conventional	-0.5			[14]
Pepper	conventional	2.2			[14]
Pepper	conventional	3.5	0.7	3	[7]
Pepper	conventional	4.7	0.9	3	[1]
Pepper	organic	7.9			[14]
Pepper	organic	14.5	1.2	2	[1]
Pepper	conventional	8.72			[15]
Pepper	organic	11.16			[15]
Perilla ocymoides L	conventional	4.5		1	[1]
Perilla ocymoides L	organic	19.9		1	[1]
Potato	conventional	2.23		9	[16]
Potato	conventional	3.76		10	[16]
Potato	conventional	4.13		10	[16]
Potato	conventional	3.23		10	[16]
Potato	conventional	0.9		3	[2]
Potato	organic	5.7		10	[16]
Potato	organic	6.99		9	[16]

Potato	organic	10.34		10	[16]
Potato	organic	5.68		10	[16]
Potato	organic	4.3		3	[2]
Potato	conventional	3.8	1.1	4	[7]
Potato	organic	8.4	2.2	3	[7]
Pumpkin	organic	5.7		3	[2]
Pumpkin	conventional	3.5		3	[2]
Rice	conventional	3.0	0.3	3	[17]
Rice	conventional	3.5	0.3	3	[17]
Rice	conventional	4.1	1.2	3	[17]
Rice	conventional	4.11		5	[18]
Rice	conventional	3.87	0.89	60	[19]
Rice	organic	4.9	0.3	3	[17]
Rice	organic	5.5	0.1	3	[17]
Rice	organic	4.8	0.1	3	[17]
Rice	organic	6.02		5	[18]
Rice	organic	6.07	0.65	60	[19]
Rocket	conventional	1.1	1.6	3	[7]
Rocket	organic	7.2	4.1	5	[7]
Sesame	conventional	5.7		1	[1]
Sesame	organic	17.8	4.8	2	[1]
Spinach	conventional	3.05	0.94	22	[3]
Spinach	conventional	5.6	1.1	2	[1]
Spinach	organic	12.20	1.26	20	[3]
Spinach	organic	11.06	1.53	20	[3]
Spinach	organic	7.20	1.66	14	[3]
Spinach	organic	9.5	0.2	2	[1]
Strawberry	conventional	2.5		82	[9] data from [10]
Strawberry	organic	3.5		82	[9] data from [10]
Tomato	conventional	-1.2		3	[2]
Tomato	conventional	-0.7		3	[2]
Tomato	conventional	7.8		3	[2]

Tomato	conventional	2.09	0.78	6	[20]
Tomato	organic	8.1	3.2	61	[6]
Tomato	organic	6.9		3	[2]
Tomato	conventional	-0.1	2.1	46	[6]
Tomato	conventional	-2.4			[14]
Tomato	conventional	0			[14]
Tomato	conventional	1.19	1.71	5	[3]
Tomato	conventional	1.28	1.34	5	[3]
Tomato	conventional	4.4	3.2	3	[7]
Tomato	conventional	0.92	0.30	6	[20]
Tomato	conventional	1.34	0.23	6	[20]
Tomato	conventional	0.24	0.04	6	[20]
Tomato	conventional	0.43	0.11	6	[20]
Tomato	conventional	0.32	0.08	6	[20]
Tomato	conventional	-2.5	2.0		[8] data from [5]
Tomato	conventional	0.3	0.6		[21] data from [5]
Tomato	organic	7.3		3	[2]
Tomato	organic	9.3			[14]
Tomato	organic	5.57	2.66	20	[3]
Tomato	organic	0.70	0.59	2	[3]
Tomato	organic	4.88	0.97	4	[3]
Tomato	organic	7.82	2.16	5	[3]
Tomato	organic	8.0	1.3	3	[7]
Tomato	organic	5.46	0.90	6	[20]
Tomato	organic	2.63	0.43	6	[20]
Tomato	organic	2.35	0.54	6	[20]
Tomato	organic	1.28	0.15	6	[20]
Tomato	organic	1.61	0.18	6	[20]
Tomato	organic	0.90	0.07	6	[20]
Tomato	organic	5.9	2.0		[8] data from [5]
Tomato	organic	7.1	0.7		[21] data from [5]
Wheat	organic	7.3	0.6	2	[22]

Wheat	conventional	2.3	1.0		[14]
Wheat	conventional	1.4	0.4	2	[22]
Wheat	conventional	1.2	0.3	2	[22]
Wheat	conventional	1.1	0.1	2	[22]
Wheat	organic	3.6	1.6		[14]
Wheat	organic	5.4	0.3	2	[22]
Wheat	organic	7.3	0.1	2	[22]
Wheat	organic	2.6	0.4	2	[22]
Wheat	organic	0.8	0.4	2	[22]
Wheat	organic	0.5	0.3	2	[22]
Zucchini	conventional	2.4		3	[2]
Zucchini	organic	10.6		3	[2]

SD, standard deviation.

Text S1. Calculations of the effect of organic food intake on tissue proteins $\delta^{15}N$.

In order to relate the $\delta^{15}N$ of tissue proteins ($\delta^{15}N_{tissue\ proteins}$) to the $\delta^{15}N$ of consumed animal proteins ($\delta^{15}N_{animal}$) and plant proteins ($\delta^{15}N_{plants}$), a two-sources isotope mixing model was used:

$$\delta^{15}N_{tissue\ proteins} = \delta^{15}N_{animal} \times P_{animal} + \delta^{15}N_{plants} \times (1\text{-}P_{animal}) + \Delta^{15}N_{tissue\text{-}diet} \quad (1)$$

where $\delta^{15}N_{animal}$ and $\delta^{15}N_{plants}$ are weighted by P_{animal} and $1\text{-}P_{animal}$, the proportions of dietary proteins occupied by animal and plant proteins, respectively (and where protein refers to protein nitrogen). $\Delta^{15}N_{tissue\text{-}diet}$ is the isotopic offset between tissue and dietary proteins, often referred to as the trophic step or discrimination factor. The effect of organic plant intake on $\delta^{15}N_{plants}$ was then similarly calculated with a two-sources isotope mixing model accounting for organic and conventional plants intake:

$$\delta^{15}N_{plants} = \delta^{15}N_{org\ plants} \times P_{org} + \delta^{15}N_{conv\ plants} \times (1\text{-}P_{org}) \quad (2)$$

where $\delta^{15}N_{org\ plants}$ and $\delta^{15}N_{conv\ plants}$ are the weighted average of the $\delta^{15}N$ of consumed organically and conventionally grown plants, respectively. P_{org} is the proportion of dietary plant proteins occupied by proteins from organically grown plants and 1- P_{org} is the proportion occupied by conventionally grown plants (and where protein refers to protein nitrogen). Rearranging equation 2 and substituting it into equation 1 yields the following equation:

$$\delta^{15}N_{tissue\ proteins} = \delta^{15}N_{animal} \times P_{animal} + [\ \delta^{15}N_{conv\ plants} + (\delta^{15}N_{org\ plants} - \delta^{15}N_{conv\ plants}) \times P_{org}\ ] \times (1\text{-}P_{animal}) + \Delta^{15}N_{tissue\text{-}diet} \quad (3)$$

The difference between $\delta^{15}N_{org\ plants}$ and $\delta^{15}N_{conv\ plants}$ has already been reported in the literature using the capital delta notation ($\Delta^{15}N_{org\text{-}conv} = \delta^{15}N_{org\ plants} - \delta^{15}N_{conv\ plants}$), therefore to simplify the equation, this notation was substituted into equation 3:

$$\delta^{15}N_{tissue\ proteins} = \delta^{15}N_{animal} \times P_{animal} + (\delta^{15}N_{conv\ plants} + \Delta^{15}N_{org\text{-}conv} \times P_{org}) \times (1\text{-}P_{animal}) + \Delta^{15}N_{tissue\text{-}diet} \quad (4)$$

When organic plants with a higher $\delta^{15}N$ than conventionally grown plants are consumed, the effect on $\delta^{15}N_{tissue\ proteins}$ can be calculated and is referred here as the error. The absolute error is the difference between the actual measured value obtained with equation 4 and the expected values when no organic plants are consumed, which is also obtained with equation 4, but with P_{org} set at 0. Subtracting these two equations gives:

$$\delta^{15}N\ \text{absolute error} = \Delta^{15}N_{org\text{-}conv} \times P_{org} \times (1\text{-}P_{animal}) \quad (5)$$

The relative error was then calculated by dividing the absolute error by the expected value when no organic plants are consumed, obtained from equation 4 with P_{org} set at 0:

$$\delta^{15}N\ \text{relative error} = 100 \times \Delta^{15}N_{org\text{-}conv} \times P_{org} \times (1\text{-}P_{animal})\ /\ [\ \delta^{15}N_{animal} \times P_{animal} + \delta^{15}N_{conv\ plants} \times (1\text{-}P_{animal}) + \Delta^{15}N_{tissue\text{-}diet}\ ] \quad (6)$$

References

1. Choi, W.-J.; Ro, H.-M.; Hobbie, E.A. Patterns of natural 15N in soils and plants from chemically and organically fertilized uplands. Soil Biol. Biochem. 2003, 35, 1493–1500.
2. Rogers, K.M. Nitrogen isotopes as a screening tool to determine the growing regimen of some organic and nonorganic supermarket produce from New Zealand. J. Agric. Food Chem. 2008, 56, 4078–4083.
3. Verenitch, S.; Mazumder, A. Isotopic characterization as a screening tool in authentication of organic produce commercially available in western North America. Isot. Environ. Health Stud. 2015, 51, 332–343.
4. Yuan, Y.; Zhao, M.; Zhang, Z.; Chen, T.; Yang, G.; Wang, Q. Effect of different fertilizers on nitrogen isotope composition and nitrate content of Brassica campestris. J. Agric. Food Chem. 2012, 60, 1456–1460.
5. Inácio, C.T.; Chalk, P.M.; Magalhães, A.M. Principles and limitations of stable isotopes in differentiating organic and conventional foodstuffs: 1. Plant products. Crit. Rev. Food Sci. Nutr. 2015, 55, 1206–1218.
6. Bateman, A.S.; Kelly, S.D.; Woolfe, M. Nitrogen isotope composition of organically and conventionally grown crops. J. Agric. Food Chem. 2007, 55, 2664–2670.
7. Šturm, M.; Lojen, S. Nitrogen isotopic signature of vegetables from the Slovenian market and its suitability as an indicator of organic production. Isot. Environ. Health Stud. 2011, 47, 214–220.
8. Bateman, A.S.; Kelly, S.D.; Jickells, T.D. Nitrogen isotope relationships between crops and fertilizer: Implications for using nitrogen isotope analysis as an indicator of agricultural regime. J. Agric. Food Chem. 2005, 53, 5760–5765.
9. Camin, F.; Perini, M.; Bontempo, L.; Fabroni, S.; Faedi, W.; Magnani, S.; Baruzzi, G.; Bonoli, M.; Tabilio, M.; Musmeci, S. Potential isotopic and chemical markers for characterising organic fruits. Food Chem. 2011, 125, 1072–1082.
10. Huelsemann, F.; Koehler, K.; Braun, H.; Schaenzer, W.; Flenker, U. Human dietary δ15N intake: Representative data for principle food items. Am. J. Phys. Anthropol. 2013, 152, 58–66.
11. Šturm, M.; Kacjan-Maršić, N.; Lojen, S. Can δ15N in lettuce tissues reveal the use of synthetic nitrogen fertiliser in organic production? J. Sci. Food Agric. 2011, 91, 262–267.
12. Szpak, P.; Millaire, J.-F.; White, C.D.; Longstaffe, F.J. Influence of seabird guano and camelid dung fertilization on the nitrogen isotopic composition of field-grown maize (Zea mays). J. Archaeol. Sci. 2012, 39, 3721–3740.
13. Rapisarda, P.; Camin, F.; Fabroni, S.; Perini, M.; Torrisi, B.; Intrigliolo, F. Influence of different organic fertilizers on quality parameters and the δ15N, δ13C, δ2H, δ34S, and δ18O values of orange fruit (Citrus sinensis L. Osbeck). J. Agric. Food Chem. 2010, 58, 3502–3506.
14. Schmidt, H.; Roßmann, A.; Voerkelius, S.; Schnitzler, W.H.; Georgi, M.; Graßmann, J.; Zimmermann, G.; Winkler, R. Isotope characteristics of vegetables and wheat from conventional and organic production. Isot. Environ. Health Stud. 2005, 41, 223–228.
15. del Amor, F.M.; Navarro, J.; Aparicio, P.M. Isotopic discrimination as a tool for organic farming certification in sweet pepper. J. Environ. Qual. 2008, 37, 182–185.
16. Camin, F.; Moschella, A.; Miselli, F.; Parisi, B.; Versini, G.; Ranalli, P.; Bagnaresi, P. Evaluation of markers for the traceability of potato tubers grown in an organic versus conventional regime. J. Sci. Food Agric. 2007, 87, 1330–1336.
17. Yuan, Y.; Zhang, W.; Zhang, Y.; Liu, Z.; Shao, S.; Zhou, L.; Rogers, K.M. Differentiating organically farmed rice from conventional and green rice harvested from an experimental field trial using stable isotopes and multi-element chemometrics. J. Agric. Food Chem. 2018, 66, 2607–2615.
18. Chung, I.-M.; Kim, J.-K.; An, Y.-J.; Kwon, C.; Kim, S.-Y.; Yang, Y.-J.; Yarnes, C.T.; Chi, H.-Y.; Kim, S.-H. Compound-specific δ13C and δ15N analyses of fatty acids and amino acids for discrimination of organic, pesticide-free, and conventional rice (Oryza sativa L.). Food Chem. 2019, 283, 305–314.
19. Chung, I.-M.; Park, S.-K.; Lee, K.-J.; An, M.-J.; Lee, J.-H.; Oh, Y.-T.; Kim, S.-H. Authenticity testing of environment-friendly Korean rice (Oryza sativa L.) using carbon and nitrogen stable isotope ratio analysis. Food Chem. 2017, 234, 425–430.
20. Pieper, J.R.; Barrett, D.M. Effects of organic and conventional production systems on quality and nutritional parameters of processing tomatoes. J. Sci. Food Agric. 2009, 89, 177–194.
21. Nakano, A.; Uehara, Y.; Yamauchi, A. Effect of organic and inorganic fertigation on yields, δ 15 N values, and δ 13 C values of tomato (Lycopersicon esculentum Mill. cv. Saturn). Plant and Soil 2003, 255, 343–349, doi:10.1023/A:1026180700963.

22. Laursen, K.H.; Mihailova, A.; Kelly, S.; Epov, V.; Bérail, S.; Schjørring, J.K.; Donard, O.; Larsen, E.H.; Pedentchouk, N.; Marca-Bell, A. Is it really organic?—Multi-isotopic analysis as a tool to discriminate between organic and conventional plants. Food Chem. 2013, 141, 2812–2820.

© 2020 by the authors. Submitted for possible open access publication under the terms and conditions of the Creative Commons Attribution (CC BY) license (http://creativecommons.org/licenses/by/4.0/).
